# Nasal Turbinate Mesenchymal Stromal Cells Preserve Characteristics of Their Neural Crest Origin and Exert Distinct Paracrine Activity

**DOI:** 10.3390/jcm10081792

**Published:** 2021-04-20

**Authors:** Hyun-Jee Kim, Sungho Shin, Seon-Yeong Jeong, Sun-Ung Lim, Dae-Won Lee, Yunhee-Kim Kwon, Jiyeon Kang, Sung-Won Kim, Chan-Kwon Jung, Cheolju Lee, Il-Hoan Oh

**Affiliations:** 1 Catholic High-Performance Cell Therapy Center, Department of Medical Lifescience, College of Medicine, The Catholic University, Seoul 06591, Korea; kimhj92@catholic.ac.kr (H.-J.K.); asklpios@hotmail.com (S.-Y.J.); woong@catholic.ac.kr (S.-U.L.); bluenimo@naver.com (D.-W.L.); acme807@gmail.com (J.K.); 2 Center for Theragnosis, Korea Institute of Science and Technology, Seoul 02792, Korea; sungho@kist.re.kr (S.S.); clee270@kist.re.kr (C.L.); 3 Department of Biology, Kyung-Hee University, Seoul 02453, Korea; kimyh@khu.ac.kr; 4 Department of Otolaryngology-Head and Neck Surgery, Seoul St. Mary’s Hospital, The Catholic University, Seoul 06591, Korea; kswent@catholic.ac.kr; 5 Department of Pathology, Seoul St. Mary’s Hospital, The Catholic University, Seoul 06591, Korea; ckjung@catholic.ac.kr

**Keywords:** turbinate MSC, neural crest, secretome

## Abstract

The sources of mesenchymal stromal cells (MSCs) for cell therapy trials are expanding, increasing the need for their characterization. Here, we characterized multi-donor, turbinate-derived MSCs (TB-MSCs) that develop from the neural crest, and compared them to bone marrow-derived MSCs (BM-MSCs). TB-MSCs had higher proliferation potential and higher self-renewal of colony forming cells, but lower potential for multi-lineage differentiation than BM-MSCs. TB-MSCs expressed higher levels of neural crest markers and lower levels of pericyte-specific markers. These neural crest-like properties of TB-MSCs were reflected by their propensity to differentiate into neuronal cells and proliferative response to nerve growth factors. Proteomics (LC–MS/MS) analysis revealed a distinct secretome profile of TB-MSCs compared to BM and adipose tissue-derived MSCs, exhibiting enrichments of factors for cell-extracellular matrix interaction and neurogenic signaling. However, TB-MSCs and BM-MSCs exhibited comparable suppressive effects on the allo-immune response and comparable stimulatory effects on hematopoietic stem cell self-renewal. In contrast, TB-MSCs stimulated growth and metastasis of breast cancer cells more than BM-MSCs. Altogether, our multi-donor characterization of TB-MSCs reveals distinct cell autonomous and paracrine properties, reflecting their unique developmental origin. These findings support using TB-MSCs as an alternative source of MSCs with distinct biological characteristics for optimal applications in cell therapy.

## 1. Introduction 

Mesenchymal stromal cells (MSCs) are non-hematopoietic adherent cell populations with multi-lineage differentiation potential towards diverse types of tissues, including bone, cartilage, vessels, and neuron-like cells [1,2,3], and exhibits common surface phenotypes [4]. Cell therapeutic trials utilizing ex vivo expanded MSCs are increasing, and their potential within a wide range of applications is being explored. These include the regeneration of damaged cardiovascular [5], neural [6], and muscular–skeletal tissues [7,8,9,10]; the suppression of allogenic immune reactions [11,12], and facilitation of hematopoietic engraftment [13,14] or suppression of graft versus host diseases [15,16].

However, significant heterogeneity in the morphology, proliferation, and differentiation potentials of MSCs has been observed during their culture [17,18,19]. The heterogeneity of MSCs can also originate from differences in the ontological stage [20] or differences in the tissue source [21,22,23]. Accumulating studies have shown that the heterogeneity of MSCs can cause a difference in the biological properties and cell therapeutic outcomes [20,21,22,23,24]. Moreover, the sources of MSCs in the cell therapy trials are rapidly expanding, and includes bone marrow (BM), adipose tissue, placenta, and Warton–Jelly derived MSCs. Accordingly, with these increasing sources and their heterogeneity, the precise characterization of MSCs is needed in order to optimize the choice of MSCs for a specific therapeutic application based on their distinct biological characteristics.

Recently, MSCs from the olfactory epithelium or nasal turbinate are emerging as an alternative source of MSCs that originates from the neural crest, unlike MSCs from conventional sources developed from the mesodermal origin [25,26,27]. Previous studies have shown that the neural crest-derived stem cells (NCSC) harbor extensive plasticity for serving as multipotent stem cells/progenitors [28] that can differentiate into multiple lineages [29], including MSCs in the cranial regions and other organs [30].

However, the potential distinctions in the biological roles of TB-MSCs as neural crest-derived MSCs as opposed to mesoderm-origin MSCs remains unclear. Therefore, in this study, we investigated the possibility that the NCSC-derived MSCs obtained in the turbinate exhibit distinct cellular characteristics and paracrine functions and cause distinct biological effects in MSC-based cell therapeutic trials. Our study should aid in the optimization of MSC source selection with respect to their developmental origin.

## 2. Materials and Methods

### 2.1. Isolation of Cells and Culture

Umbilical cord blood was obtained from the Seoul Metropolitan Government Public Cord Blood Bank. BM-MSCs from bone marrow aspirates or TB-MSCs from nasal turbinate were obtained under informed consent from healthy donors or patients with nasal septal deviation, respectively, under approval from the Institutional Review Board of the Catholic University of Korea and Seoul St. Mary’s Hospital, the Catholic University of Korea (MC19TNSI0012 and KC08TISS0341). MSC cultures were established from the mononuclear cell fraction and maintained in Dulbecco’s modified Eagle’s medium (DMEM; Hyclone Laboratories Inc, Schenectady, NY, USA) containing 10% fetal bovine serum (FBS; Hyclone, Logan, UT, USA), L-glutamine, 100 U/mL penicillin, and 100 mg/mL streptomycin in a humidified 5% CO_2_ at 37 °C.

### 2.2. Phenotyping of MSCs

MSCs were analyzed for phenotype by flowcytometry using monoclonal antibodies against human CD34, CD90, CD146, CD140b, CD13, CD19, HLA-DR, CD14, CD45, CD105, and CD73 (BD Pharmingen, San Jose, CA, USA) and anti-human CD166 (Serotec, Oxford, UK), according to the manufacturer’s instructions. 

### 2.3. Colony-Formation Assay

For colony formation of MSCs (CFU-F), MSCs were plated in a dish (300 cells per 80 cm^2^; 100 mm dish) and incubated for 14 days, and then the number of colonies containing >50 cells were counted by staining with crystal violet (Sigma, St. Louis, MO, USA) in methanol, as described [31]. The secondary CFU-F was counted by replating the primary CFU-F for 2 weeks.

### 2.4. Multilineage Differentiation Assays

Osteogenic differentiation was induced by DMEM supplemented with 10% FBS, 10 nM dexamethasone, 0.2 mM ascorbic acid, and 10 mM β-glycerophosphate. After 14 days, the mineralization of the extracellular matrix was determined by Alizarin Red S staining, and extraction with 10% cetylpyridinium chloride followed by measurement of the absorbance at 550 nm. For adipogenic differentiation, confluent cells were incubated in DMEM containing 10% FBS, 1 µM dexamethasone, 0.5 mM isobutyl-1-methylxanthine, 100 µM indomethacin, and 10 µg/mL insulin. After 14 days, cells were fixed with propylene glycol and stained with Oil Red O to visualize lipid droplets. The numbers of lipid droplets were subsequently determined by dye extraction using 4% Nonidet P40 in isopropyl alcohol followed by spectrophotometry at 520 nm.

### 2.5. RNA Extraction and qRT-PCR

Total RNA from MSC was isolated with Trizol (Invitrogen, San Diego, CA, USA). cDNA was synthesized from l–2 µg of total RNA with superiorScript III mastermix (Enzynomics, Daejeon, Korea). mRNA levels of *SOX2, OCT4, NANOG, PAX3, CD13, CD146, PDGFRβ, NG2*, *NESTIN, SYP (synaptophysin)*, and *PAX3* were measured by quantitative real-time PCR (qRT-PCR) using SYBR Premix Ex Taq II (TaKaRa, Shiga, Japan). Normalization and fold changes were calculated using the 2^−ΔΔCt^ method using GAPDH as internal control. 

### 2.6. Growth Factors for Cell Stimulation

For stimulation of cell growth, MSCs in the exponential growth phase were treated with recombinant human β-NGF (rNGF) (100 ng/mL) (PeproTech, Rocky Hill, NJ, USA) and recombinant bFGF (100 ng/mL) (PeproTech) for 3 days, and proliferation was determined by fold increase in cell numbers. Effects on MSC phenotype were analyzed by flow cytometry using antibody against human CD271 (BD Pharmingen).

### 2.7. Co-Culture

Human CD34^+^ cells were purified from umbilical cord blood using a CD34 MicroBead kit (Miltenyl Biotec, Bergisch Gladbach, Germany). For co-culture, MSCs were pretreated with mitomycin-C (10 µg/mL) for 1 h, washed, and co-cultured with purified CD34^+^ cells for 4 days in DMEM + 10% FBS (Hyclone) in the presence of cytokine mixtures (20 ng/mL hSCF, 20 ng/mL hFlt3L, 4 ng/mL hIL-3, 4 ng/mL hIL-6, 4 ng/mL hG-CSF, and 0.2 × 10^−6^ M hydrocortisone (ProSpec-Tany TechnoGene Ltd., Rehovot, Israel). For phenotypic analysis of ex vivo expanded hematopoietic cells, co-cultured cells were stained with antibodies against CD45, CD34, and CD90 (BD Pharmingen, San Jose, CA, USA) and analyzed by flow cytometry after gating the hematopoietic (CD45^+^) population. Colony-forming cell (CFC) assays were performed by plating the co-cultured cells in semisolid medium (H4230; STEMCELL Technologies, Vancouver, Canada) for 14 days.

### 2.8. Neural Differentiation

For neural differentiation of MSCs, cells were plated in a fibronectin-precoated dish (60–80% confluency) and induced to differentiate into neuronal cells for 3 or 7 days using the neuronal induction media (PromoCell-C28015, Heidelberg, Germany) with medium changes in every 48 h.

### 2.9. Immunocytochemistry and Western Blotting

MSCs were plated in fibronectin-coated chamber slides and fixed with cold methanol. For blocking, cells were incubated in 10% goat serum-containing PBS for 1 h at room temperature. After washing, the chamber slides were incubated with primary antibody against Synaptophysin (SYP) (Abcam, Cambridge, MA, USA), and visualized by secondary antibody. Fluorescent images were obtained using a confocal laser scanning microscope (LSM700; ZEISS International, Oberkochen, Germany) and analyzed by using ZEISS ZEN Imaging Software. Expression of neuronal markers was also analyzed by Western blots using antibodies against neurofilament (NF), NEUN, MAP2, and NESTIN (Abcam) and a Luminescent Image Analysis System (LAS-4000; Fuji film, Tokyo, Japan) with quantification using Image J software.

### 2.10. Tumor Growth and Metastasis

The human breast cancer cell line MDA-MB-231 was purchased from ATCC (Manassas, VA, USA). Animal experiments were approved by the Catholic University of Korea Animal Care and Use Committee (IACUC no. 2018-0172-01). MDA-MB-231 cells (5 × 10^5^) and MSCs from TB or BM (5 × 10^5^) were co-injected into mammary fat pads of immune-deficient NOD scid gamma (NSG) mice in a mixture with growth factor-reduced Matrigel (BD). Primary tumor volumes were measured as previously described (width × length × height × 0.52) [32]. Distant metastasis was analyzed by gross and microscopic analysis of organs by hematoxylin/eosin staining.

### 2.11. Immune Suppression Assay

Mononuclear cells (MNCs) from human umbilical cord blood were stained with CFSE using a CellTrace CFSE cell proliferation kit (5 μM, Thermo Fisher Scientific, Waltham, MA, USA) according to the manufacturer’s protocol. The MNCs were mixed with mitomycin-C pre-treated MSCs and stimulated by anti-CD3/CD28 microbeads (Gibco) and recombinant human IL-2 (30 U/mL, PeproTech). The proliferation of T-cells was measured by flow cytometry analysis for decrease of CFSE intensity in the gated (CD45^+^CD3^+^CD8^+^ or CD45^+^CD3^+^CD4^+^) subsets of T-cells.

### 2.12. Mass Spectrometric (MS) Analysis of Secreted Proteins

The collected secretome samples (100 μg protein) were analyzed by nano-flow liquid chromatography tandem mass spectrometry (LC–MS/MS) on an LTQ-Orbitrap XL mass spectrometer (Thermo Fisher Scientific) after tryptic digestion. The detailed procedure is the same as described in our previous paper [33], except that the length of the analytical column was 15 cm. Each biological replicate of the secretome was analyzed in a single LC–MS/MS run.

### 2.13. Bioinformatic Analysis of Mass Spectrometric Data

The mass spectrometric data were loaded on the Proteome Discoverer (ver2.2.0.388) software, and label-free quantification (LFQ) was performed to compare the expression levels of each individual protein between replicates and between different MSCs. We used the human UniProtKB database (released in June 2020) with the FBS protein list added for the MS data search to exclude suspected proteins affected by FBS [34]. Only the proteins confirmed as true secretory proteins by either SignalP [35], SecretomeP (score ≥ 0.5) [36], or TMHMM [37] were used for the next step of gene ontology (GO) analysis. GO terms were analyzed using the algorithm of the Database for Annotation, Visualization, and Integrated Discovery (DAVID) tools (https://david.ncifcrf.gov/, accessed on 19 March 2021). The prediction of biological functions and upstream regulators were analyzed using Ingenuity Pathway Analysis (IPA) software (content version: 47547484). A *t*-test for LFQ intensity values was performed, and proteins with *p*-values less than 0.05 were used for IPA analysis. Uploaded data for upstream regulator analysis contained UniProtKB accession and the log2 ratio of identified proteins. Predicted upstream regulators with a Z-score above 2 and a *p*-value of overlap below 0.01 were considered significantly activated.

### 2.14. Statistical Analysis

Two-group comparisons were conducted using a two-tailed, unpaired *t*-test. Data were expressed as mean ± standard error of the mean (SEM). Significance levels were indicated as * *p* < 0.05, ** *p* < 0.01, and *** *p* < 0.001. One-way ANOVA was used to analyze the differences among the means for three or more groups. When ANOVA test showed significant difference between the groups, Tukey’s test was used as post hoc tests to find differences between specific groups. Significance levels were indicated as * *p* < 0.05. Statistical analysis was performed using GraphPad Prism software (version 8.0.1; GraphPad Software, San Diego, CA, USA).

## 3. Results

### 3.1. Turbinate and Bone Marrow MSCs Exhibit Distinct Cellular Characteristics

**Figure 1:**
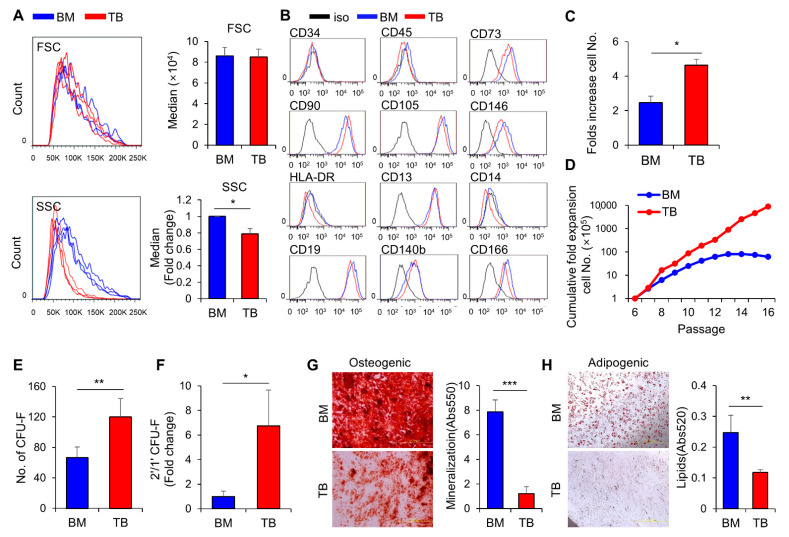
Biological characteristics of turbinate-derived MSCs (TB-MSCs) compared with BM-derived MSCs (BM-MSCs). (**A**) Representative flow cytometry profiles of TB and BM-MSCs for cell size (FSC) and density (SSC) (left) and quantitative analysis of the median values (right) (mean ± SEM, * *p* < 0.05, three expts, *n* = 3). (**B**) Flow cytometry analysis of TB and BM-MSCs for canonical phenotypic markers of MSCs. (**C**) Proliferation potential of TB and BM-MSCs. Cells were seeded at the same density (2.5 × 10^3^/cm^2^), and cell numbers counted after 3 days of culture. Shown are the numbers of cells after the culture period for each of three individual donor-derived TB-MSCs and BM-MSCs (mean ± SEM, * *p* < 0.05, six expts, *n* = 3). (**D**) Replicative senescence of TB and BM-MSCs. Each MSC type was plated at identical density (3.5 × 10^3^/cm^2^; 100 mm dish) and subcultured in growth media with serial subcultures. Shown are the long-term growth potentials over the indicated passages for TB and BM-MSCs (one expt, *n* = 2 for each). (**E**) The frequency of CFU-F for BM and TB-MSCs. Cells in the exponential growth phase were plated at the same density (300 cells/100 mm plate), and formed colonies were identified by crystal violet staining. Shown are the mean numbers of CFU-F from three independent donors of TB and BM-MSCs (mean ± SEM, ** *p* < 0.01, three expts, *n* = 12). (**F**) Self-renewal potential of colony-forming cells (CFU-F) in TB and BM-MSCs. Colonies formed in each plate were harvested and re-plated for self-renewal. Shown are the ratios of secondary/primary colony numbers obtained from three individual donors of TB and BM-MSCs (mean ± SEM, * *p* < 0.05, two expts, *n* = 12). (**G**,**H**) Multi-lineage differentiation potential of TB and BM-MSCs. Each MSC type was subjected to osteogenic and adipogenic differentiation. Shown are representative images of terminal differentiation (left) and quantification of osteogenic (**G**) or adipogenic (**H**) differentiation from three individual donors of TB and BM-MSCs as determined by droplets (Abs 520 nm) and mineralization (Abs 550 nm), respectively (mean ± SEM, ** *p* < 0.01, *** *p* < 0.001, three expts, *n* = 9).

To compare MSCs derived from the nasal turbinate (TB-MSCs) with bone marrow-derived MSCs (BM-MSCs), and to control for individual variation, we collected each tissue-source of MSCs from multiple donors at similar ages and compared their biological properties. TB-MSCs exhibited a similar cell size as BM-MSCs, but their cell density was lower, as evidenced by the comparable forward scatter (FSC) and lower side scatter (SSC) in flow cytometry (Figure 1A). The surface phenotypes of TB-MSCs were also similar to those of BM-MSCs, exhibiting a common canonical phenotype for MSCs (Figure 1B).

We next compared the growth kinetics of TB-MSCs to BM-MSCs. As shown in Figure 1C, TB-MSCs exhibited a higher proliferative activity than BM-MSCs. Moreover, TB-MSCs exhibited a delay in replicative senescence; TB-MSCs continued to grow beyond passage 16, whereas BM-MSCs reached growth arrest after 11–13 passages (Figure 1D). When examined for colonogenic potential, TB-MSCs exhibited a significantly higher frequency of CFU-F than BM-MSCs (Figure 1E). Moreover, the numbers of secondary CFU-F after replating the primary colonies were strikingly higher in TB-MSCs than for BM-MSCs, indicating higher self-renewal of colonogenic MSC progenitors (CFU-F) in TB-MSCs (Figure 1F). In contrast, when examined for multi-lineage differentiation potential into osteogenic or adipogenic lineages, TB-MSCs exhibited decreased terminal differentiation into osteogenic and adipogenic lineages compared to BM-MSCs (Figure 1G,H).

Together, these results reveal that TB-MSCs exhibit higher proliferative potential with higher self-renewal rates of colonogenic progenitors, but lower potential for multi-lineage differentiation than BM-MSCs.

### 3.2. Turbinate MSCs Preserve the Neural Crest-Like Properties in Cell Autonomous Effects

**Figure 2:**
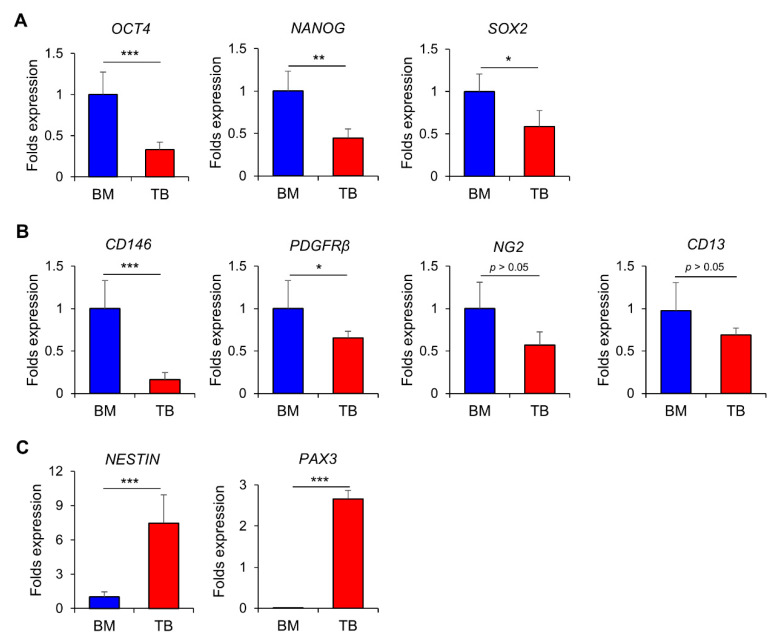
Cell fate characterization of TB and BM-MSCs by gene expression patterns. TB and BM-MSCs from three individual donors were examined for the expression of genes related to pluripotency (**A**), pericyte markers (**B**), and neural crest markers (**C**). Shown are the expression levels of each indicated gene in TB-MSCs relative to the levels in BM-MSCs (mean ± SEM, * *p* < 0.05, ** *p* < 0.01, *** *p* < 0.001, three expts, *n* = 9).

Based on these differences in the cellular properties of TB and BM-MSCs, we examined the potential differences in the expression of genes that characterize the cellular identity of MSCs. For pluripotency-related genes, TB-MSCs exhibited significantly lower levels of expression of *OCT4*, *NANOG*, and *SOX2*, indicating that the higher proliferation potential of TB-MSCs is not attributed to an increase in the stemness-related genes (Figure 2A). Next, we compared the expression of pericyte markers; TB-MSCs expressed lower levels of pericyte markers than BM-MSCs, including *CD146*, *PDGFRß*, *NG2*, and *CD13* [38,39] (Figure 2B). In contrast, TB-MSCs expressed strikingly higher levels of neural crest markers, such as *NESTIN* and *PAX3* [40,41] (Figure 2C). Together, these results show that TB-MSCs preserve the characteristic features of neural crest-derived stem cells, while down-regulating genes required for pluripotency and pericyte-related genes.

**Figure 3:**
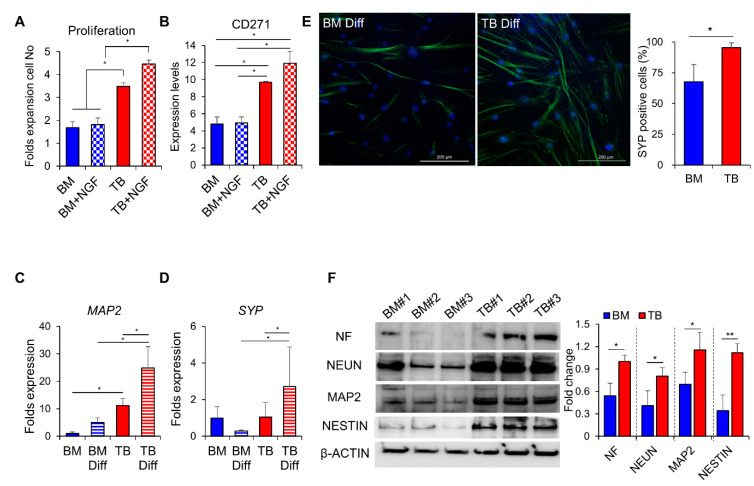
Neurogenic potential of TB-MSCs. (**A**) Response of TB and BM-MSCs to NGF. TB or BM-MSCs from three individual donors were plated in the same density (3 × 10^4^/cm^2^) and stimulated by NGF (100 ng/mL) for 3 days, and their proliferative response in the presence or absence of NGF were quantified. Shown are the increments of cell numbers in TB-MSCs relative to the increase in BM-MSCs from each individual donor (one-way ANOVA followed by Tukey’s post-test, * *p* < 0.05, two expts, *n* = 3). (**B**) Expression levels of a receptor for NGF (CD271) in TB and BM-MSCs in the presence or absence of NGF. Shown are the folds intensity of mean fluorescence (MFI) relative to the intensities of donor cell-matched isotype controls in TB and BM-MSCs derived from three individual donors (one-way ANOVA followed by Tukey’s post-test, * *p* < 0.05, two expts, *n* = 3). (**C**–**F**) TB and BM-MSCs were induced to differentiate into neurons. (**C**,**D**) Neurogenic differentiation after 3 days of induction was quantitated by transcripts for the synaptic vesicle-specific gene (*SYP*) or the neuron-specific gene (*MAP2*) (one-way ANOVA followed by Tukey’s post-test, * *p* < 0.05, three expts, *n* = 3 from 3 donors). (**E**) Neurogenic differentiation was examined by immunofluorescence for expression of SYP 7 days after differentiation induction (left, 100X magnification) with quantification of the SYP (+) cells among the DAPI stained cells in the field (right) (one expt, *n* = 3, * *p* < 0.05). (**F**) Immunoblot analysis for expression of neuron-specific genes in MSCs after neuronal differentiation for 3 days. Shown are the representative images for immunoblot (left) and quantification of the fold changes in expression levels of each indicated gene relative to β-actin, analyzed by using Image J (right) (one expt, *n* = 6, * *p* < 0.05, ** *p* < 0.01).

To further examine the neural crest-like properties of TB-MSCs, we evaluated whether the MSCs exhibit a differential response to nerve-specific growth factors. As shown, nerve growth factors (NGF) exhibited a selective stimulatory effect on TB-MSCs, but not on BM-MSCs (Figure 3A and Supplemental Figure S1A). Consistent with this finding, higher levels of the NGF receptor (CD271) were found on the cell surface of TB-MSCs than on BM-MSCs (Figure 3B). In contrast, responses to fibroblast growth factor (FGF) were not significantly different between TB and BM-MSCs (Supplemental Figure S1B), indicating that TB-MSCs mimic neuronal cells in their response to growth factors.

Based on these findings, we next examined the neurogenic potential of TB-MSCs in comparison to BM-MSCs during induced differentiation into neurons. Induction of neuronal differentiation in TB-MSCs caused higher levels of expression of *MAP2*, a marker for neurons [42], and *SYP(synaptophysin)*, a marker specific for functional synaptic vesicle protein [43,44], compared to BM-MSCs (Figure 3C–E). Similarly, there were higher expression levels of NF (neurofilament), a marker for motor neurons [45], and NEUN, a neuron-specific markers [46], as detected by immunoblot analysis in TB-MSCs than in BM-MSCs (Figure 3F).

These results together indicate that TB-MSCs preserve the properties of neural crest stem cells and are more prone to differentiate into functional neuronal cells than BM-MSCs.

### 3.3. Proteomic Distinctions of TB and BM-MSCs in Their Secreted Proteins

**Figure 4:**
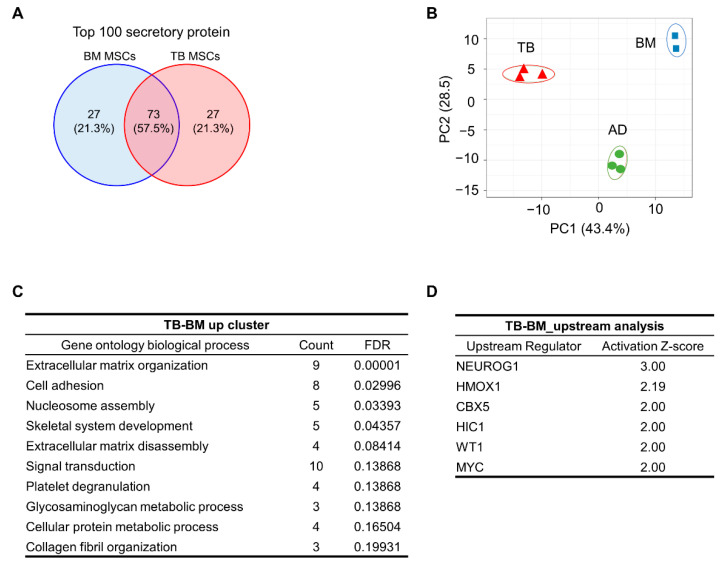
Secretome of TB and BM-MSCs. To obtain serum-free supernatants, TB and BM-MSCs grown in FBS containing media were switched into serum-free media, and the serum-free supernatants were collected 24 h after media switching. The supernatants were analyzed for secreted proteins by liquid chromatography/mass spectrophotometry (LC–MS/MS) (3 individual samples for one expt). (**A**) Shown are the % overlaps of the top 100 most abundant secreted proteins between TB and BM-MSCs. (**B**) PCA analysis of the secretome from TB-MSCs (TB), BM-MSCs (BM), and AD-MSCs (AD). (**C**) Gene ontology of secreted proteins up-regulated in TB-MSCs in comparison to BM-MSCs. Shown are the gene clusters of up-regulated proteins in supernatants of TB-MSCs compared to BM-MSCs along with counts of genes in each clusters (FDR < 0.02). (**D**) Upstream analysis of transcriptional regulators that can explain the observed increase of the secreted proteins up-regulated in supernatants of TB-MSCs relative to that of BM-MSCs. The analysis was performed using IPA software (Z-score > 2.0).

Based on these cell autonomous distinctions between TB-MSCs and BM-MSCs, we were prompted to investigate whether these MSCs could have distinct paracrine effects. For this, we first examined their secretomes by analyzing the proteins in their culture supernatants by liquid chromatography with tandem mass spectrophotometry (LC–MS/MS). The secreted proteins of TB and BM-MSCs exhibited distinctions with only about 57% similarity among the top 100 secreted proteins from each MSC (Figure 4A). These distinctions were similarly observed in the multiple PCA analyses of secreted proteins among TB-MSCs, BM-MSCs, and adipose tissue-derived MSCs (AD-MSCs), revealing a clear clustering of the TB-MSCs secretome from the other sources of MSCs (Figure 4B and Supplemental Figure S3). Gene ontology analysis of the secreted proteins enriched in TB-MSCs revealed gene clusters involved in extracellular matrix organization/disassembly and cell adhesion (Figure 4C). Similarly, analysis of disease and function prediction for the proteins enriched in TB-MSCs compared to BM-MSCs revealed significant enrichment of clusters for cancer cell growth and proliferation (Supplemental Figure S2B), suggestive of their distinctive influence on cell proliferation and cell–cell interactions. Moreover, Neurogenin1 was identified as the most significant upstream signal among the upstream signals responsible for proteins enriched in the secretome of TB-MSCs (z-score > 2.0) (Figure 4D and Supplemental Figure S2A), further supporting the neural crest-like properties of TB-MSCs. Interestingly, the distinct pattern of TB-MSCs in comparison to BM-MSCs were similarly reproduced in the comparison of TB-MSCs to AD-MSCs, exhibiting similar enrichment of proteins involved in cell adhesion and cell growth as well as enrichment of Neurogenin1 in the upstream analysis (Supplemental Figure S3A–D). These results together indicate that the secretome of TB-MSCs exhibit a conserved distinction from MSCs of mesodermal origin, including BM or AD-MSCs, further supporting the unique secretome pattern of TB-MSCs.

### 3.4. Comparisons of Paracrine Effects of TB and BM-MSCs on Normal and Cancer Stem Cells

**Figure 5:**
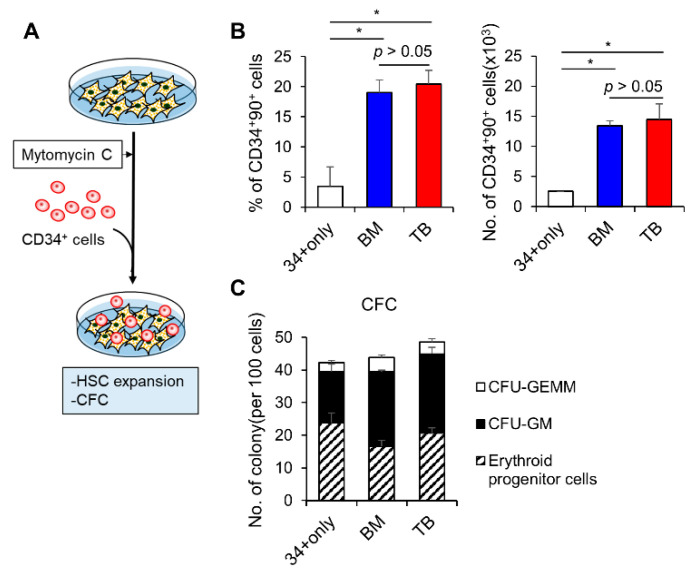
TB-MSCs were examined for HSC-supporting niche activity in comparison to BM-MSCs. (**A**) Schematic of the experimental procedure. BM or TB-MSCs (2 × 10^4^/cm^2^) were pre-treated with mitomycin-C (10 µg/mL) for growth arrest 1 h before co-culture with CD34^+^ cells (400 cells/cm^2^). Expansion of hematopoietic progenitors (CD34^+^/90^+^) or colony forming cells (CFC) after 4 days of co-culture with each group of MSCs were compared. (**B**) Expansion of primitive hematopoietic progenitors (CD34^+^90^+^) under stroma-free conditions or co-culture with each MSC type. Shown are the % of CD34^+^90^+^ subsets in hematopoietic cells (CD45^+^) (left) and numbers of CD34^+^90^+^ cells (right) after co-culture (mean ± SEM, one-way ANOVA followed by Tukey’s test, * *p* < 0.05, three expts, *n* = 9). (**C**) Colony-forming cells (CFCs) after co-culture under the indicated conditions (mean ± SEM, one-way ANOVA *p* > 0.05, three expts, *n* = 9).

To further examine the paracrine effects, we next compared the niche activities of TB-MSCs and BM-MSCs for supporting the growth and self-renewal of normal and cancer stem cells. First, to examine support for normal stem cells, TB or BM-MSCs were co-cultured with CD34^+^ hematopoietic progenitors and analyzed for the self-renewing expansion of primitive subsets of hematopoietic progenitors (CD34^+^90^+^), the subset representing BM repopulating cells [47,48] (Figure 5A). The numbers of CD34^+^90^+^ subsets were significantly increased in the co-cultures with both TB or BM-MSCs, compared to the stroma-free cultures (Figure 5B), consistent with the microenvironmental support for self-renewal of HSCs [24,49,50,51], thus indicating that the supporting activities of TB-MSCs on primitive CD34^+^90^+^ subsets were comparable to those of BM-MSCs. However, colony-forming cells (CFC) representing more down-stream precursors were not significantly increased by co-culture with TB or BM-MSCs compared to the stroma-free culture (Figure 5C). These results show TB-MSCs can similarly exert support for the self-renewing expansion of such primitive subsets of hematopoietic progenitors as BM-MSCs.

**Figure 6:**
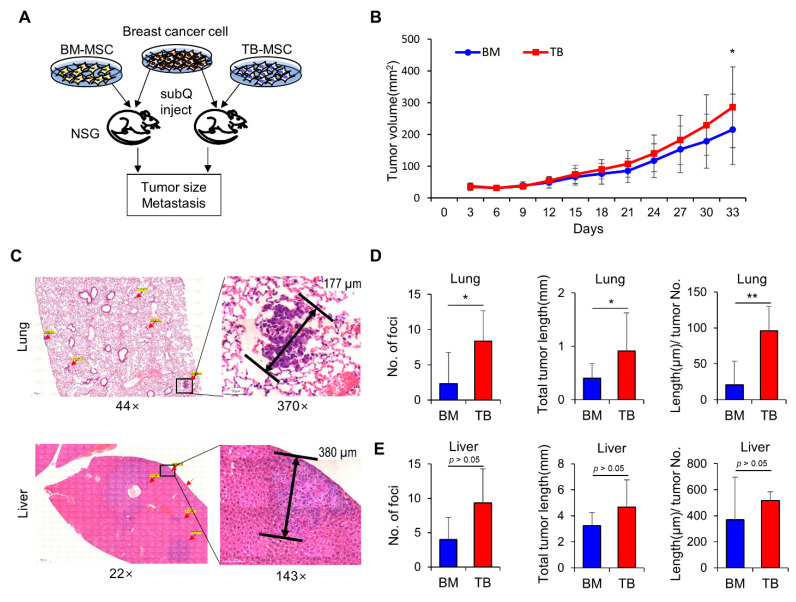
Effects of TB-MSCs and BM-MSCs on cancer cells. (**A**) Schematic of experimental procedure. Breast cancer cells (MDA-MB-231) were inoculated into the mammary fat pad of NSG mice along with BM or TB-MSCs in a mixture with Matrigel (1:1 ratio). Mice were examined for growth of primary tumor mass and distant metastasis to liver and lung. (**B**) Tumor growth of breast cancer cells. After inoculation of the tumor cells with TB or BM-MSCs, the tumor volumes were measured at the indicated time points (tumor volume (mm^2^): width × length × height × 0.52). Data from three independent donors (mean ± SEM, * *p* < 0.05, one expt, *n* = 24 for each group). (**C**–**E**) Effects of TB or BM-MSCs on metastasis of breast cancer cells. The mice with primary tumors were examined for metastasis in liver and lung by microscopy to count the number of metastatic foci and size. (**C**) Representative micrographs of metastasis by H/E staining and measurement of their length. Metastatic foci in low magnification images and their high magnification of the area indicated by box. (**D**,**E**) Quantification of metastatic foci by numbers and length in lung (**D**) and liver (**E**) (mean ± SEM, * *p* < 0.05, ** *p* < 0.01, one expt, *n* = 6 for each group).

In contrast, when TB-MSCs and BM-MSCs were examined for their effects on cancer cells by co-implanting MSCs and breast cancer cells into immune-compromised mice (NSG), TB-MSCs supported faster growth of cancer cells than did BM-MSCs, resulting in larger tumor volumes (Figure 6A,B). Moreover, the numbers of metastatic foci in lung were significantly higher in mice implanted with TB-MSCs than in mice with BM-MSCs (Figure 6C–E and Supplemental Figure S4). In addition, the length of the metastatic lesion for each metastatic tumor was also significantly higher in mice with TB-MSCs than the BM-MSC group. Given that cancer cells evolve into metastatic tumors primarily through the acquisition of stemness [52,53], these results indicate that TB-MSCs better support stemness in breast cancer cells as well as their growth in distant organs.

### 3.5. Comparisons for Immune-Modulating Effects of TB and BM-MSCs

**Figure 7:**
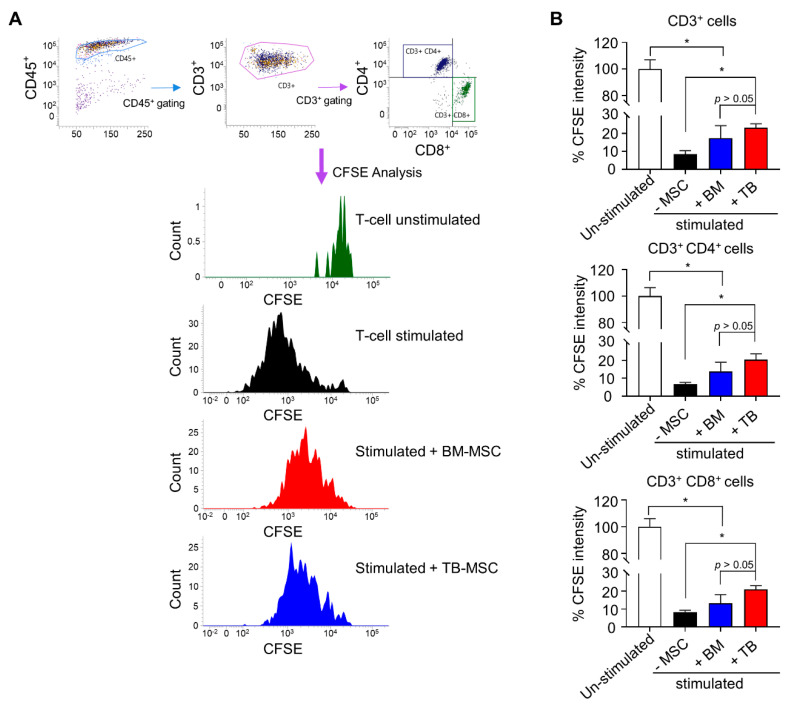
Suppression of allogenic immune responses by TB-MSCs in comparison to the BM-MSCs. (**A**) Representative flow cytometry profiles. Mononuclear cells (MNCs) from umbilical cord blood (1 × 10^5^/96 well) were stained with CFSE and stimulated with anti-CD3/CD28 microbeads and IL-2 for 6 days in the absence or presence of MSCs (1 × 10^4^/96 well). The subsequent proliferation of T-cells (CD3) and their subsets (CD3^+^CD4^+^ or CD3^+^CD8^+^) was measured by the decrease of CFSE intensity in their cell membranes. Shown are representative flow cytometry profiles for gating T-cells (CD3^+^) and their changes in CFSE intensity. (**B**) Quantitative measurement of immune suppressive function of TB and BM-MSCs. The suppression of T-cell proliferation by each type of MSCs was analyzed by the decrease in mean CFSE fluorescence intensity in CD3^+^CD4^+^ or CD3^+^CD8^+^ cells. Shown are the mean fluorescence intensity of CFSE in T-cells of each group relative to the intensity in un-stimulated group (one-way ANOVA followed by Tukey’s test, * *p* < 0.05, one expt, *n* = 3).

Given that MSCs are also frequently utilized for suppressing the immune response, we compared the immune-modulating effects of TB and BM-MSCs in suppressing the allo-immune response. Thus, mononuclear cells stained with carboxyfluorescein succinimidyl ester (CFSE), a membrane binding dye, were co-cultured with MSCs and stimulated with anti-CD3/CD28 microbeads and IL-2. As shown in Figure 7, tracking of T-cell proliferation showed that both BM and TB-MSCs inhibited the allo-immune reaction of CD4^+^ or CD8^+^ T-cells to comparable levels, indicating that TB-MSCs can also exert a comparable immune-modulating potential as BM-MSCs.

Taken together, our results reveal that TB-MSCs developed from the neural crest preserve the neural crest-like properties and exhibit a series of distinct cell autonomous as well as paracrine functions, which may have specific biological effects in MSC-based cell therapy trials.

## 4. Discussion

While MSC-based cell therapy is currently expanding into diverse clinical applications, the difference in the biological properties of MSCs and subsequent heterogeneity in clinical outcomes have been major challenges in the field.

So far, most of the heterogeneity has been thought to originate from the differences in the source of MSCs, including BM, umbilical cord blood or adipose tissue, etc., exhibiting distinct as well as common characteristics in their intrinsic biological properties [21,22,23]. However, studies have shown that additional heterogeneity of MSCs can be created during ex vivo culture, causing batch-to-batch variations in the biological function of MSCs and their outcomes from applications [54]. Similarly, differences in the ontological stage of the source tissue were also shown to cause heterogeneity in the support of normal and leukemic stem cells [20,22].

Moreover, a recent in vivo cell tracking study showed that MSCs serve different biological functions depending on their developmental origin, i.e., MSCs from the mesodermal sclerotome in BM primarily contribute to the generation of bone and adipose tissues, whereas MSCs from the neural crest contribute to Schwann cells and microenvironmental niche cells that can support the self-renewal of HSCs [41]. Given that MSCs in adipose tissue or skeletal tissues develop from somatic or visceral lateral plate mesoderm, but MSCs in the craniofacial regions are derived from the neural crest [55], it has been an unanswered question whether these different origins lead to distinct biological functions of culture expanded MSCs. In this light, the precise characterization of TB-MSCs is of particular importance, partly because they originate from the neural crest, and partly because TB-MSCs can be easily obtained without ethical conflicts from patients with nasal septum deviations, representing another important source of MSCs [25,27].

However, the quality of MSCs varies widely among donors [56,57,58], thus making it difficult to standardize the results of each individual clinical and preclinical studies [59]. To overcome these limitations, in this study, we compared TB and BM-derived MSCs with different batches from multiple donors to overcome individual variations among the same groups, and we found characteristic properties of TB-MSCs that are distinct from BM-derived MSCs. First, TB-MSCs exhibited many features that suggest preservation of the developmental memory of the neural crest. For example, TB-MSCs expressed lower levels of pluripotency or pericyte-related genes, but strikingly higher levels of genes for the neural crest, including *NESTIN* and *PAX3* [40,41]. The neural crest-like properties of TB-MSCs were similarly mirrored in their specific proliferative response to NGF. Moreover, TB-MSCs were more prone to differentiate into functional neuronal cells, expressing markers for motor neurons (NF) and synaptic vesicles (SYP), as well as neuron-specific markers (NEUN and MAP2) compared to BM-MSCs.

Of note, TB-MSCs exhibited a remarkably higher proliferation potential and self-renewing expansion of CFU-F, the mesenchymal progenitors, as well as delayed replicative senescence compared to BM-MSCs, pointing to the relative advantage of TB-MSCs for ex vivo expansion. Currently, more than 425 clinical trials have been performed using large doses of expanded MSCs (1–5 × 106/kg) [60], indicating that large-scale expansion of primary MSCs still pose a challenge in clinical trials. Moreover, primary MSCs exhibit a decline of proliferative activities with increasing age and underlying disorders [58,61]. Similarly, culture expansion of MSCs to a large number of cells are limited by continuous gene expression changes and replicative senescence [62]. In this sense, higher proliferation potential with delay in replicative senescence of TB-MSCs could open the possibility that it can serve as highly expandable MSCs without ethical compromise to overcome current limitations in cell therapy using cultured MSCs.

Interestingly, in addition to the distinction in cell autonomous properties, TB-MSCs exhibited a distinct pattern in their secreted proteins from BM-derived MSCs, which was also distinct from AD-MSCs. This indicates that the secretome of TB-MSCs could exert unique paracrine effects distinct from other MSCs. The most characteristic features of secreted proteins from TB-MSCs were the higher secretion levels of proteins involved in cell–cell interactions or cell–ECM interactions. In addition, the upstream signaling of secreted proteins revealed an enrichment of signals for Neurogenin1, the master regulatory gene for neuronal commitment [63,64], supporting their propensity to differentiate into neuronal cells. Interestingly, the distinct patten of secretome for TB-MSCs in comparison to BM-MSCs were similarly reproduced in their comparison to AD-MSCs, suggestive of a unique secretome pattern of TB-MSC, being consistently distinct from other sources of MSCs.

However, when examined for HSC supporting functions of neural crest-derived MSCs [41], TB-MSCs exhibited comparable support for self-renewing expansion of normal HSCs as BM-MSCs. These findings, given that the HSC-supporting niche activity of MSCs are controlled by various extrinsic signals [49,51], suggest that the functional distinction of MSCs with respect to their developmental origin may not be precisely reproduced in the MSCs cultured in vitro. Consistent with the possibility, MSCs from different sources were shown to exhibit differential responses to different inflammatory stimuli [21]. Thus, it is possible that while the HSC-supporting niche activities are comparable for TB and BM-MSCs under in vitro conditions, the "in vivo" niche activities under distinct extrinsic signals could be regulated in a tissue-specific manner. Further in vivo studies under physiological conditions are warranted to pursue this possibility.

In contrast, the paracrine-supporting activities on solid tumors (breast cancer cells) were distinct between the TB-MSCs and BM-MSCs; proliferation of primary tumors and their metastasis to lung were more highly stimulated by TB-MSCs than BM-MSCs. Consistent with the findings, the secretome of TB-MSCs is highly enriched with proteins whose gene function is related to cancer cell growth. Similarly, secreted proteins were most significantly enriched in TB-MSCs involved in ECM organization/disassembly and cell adhesion. Taking that the changes in cell–cell interaction or adhesion to ECM underlies the metastatic cancer cells undergoing epithelial–mesenchymal transition [65,66,67,68], it is possible that the secretome of TB-MSCs differentially influences the growth of tumor cells and the acquisition of metastatic potential. Further studies are warranted for a more comprehensive analysis of tissue-specificity in the paracrine support from TB-MSCs.

Taken together, our study shows that MSCs of neural crest origin preserve the neural crest-like nature with a commitment to neuronal differentiation along with a distinct secretion of paracrine factors resulting in distinct biological responses to environmental cues. Our findings should help rationalize the selection of different MSCs for optimization of cell therapeutic trials based on their distinct biological characteristics.

## 5. Conclusions

TB-MSCs developed from the neural crest can be easily obtained without ethical issues and can be expanded to a larger amount than BM-MSCs due to higher proliferation and self-renewing potential. TB-MSCs also preserve the developmental memories on the neural crest origin, exhibiting higher response to nerve growth factors and higher propensity to differentiation into neuronal cells. Moreover, TB-MSCs secrete distinct spectrums of proteins from BM or AD-MSCs to exert a distinct effect on cancer stem cells. Nevertheless, TB-MSCs exert comparable support on normal HSCs and comparable immune modulating effects as BM-MSCs. These comparable but distinct properties of TB-MSCs indicate that they can be utilized as alternative sources of MSCs for specific indication of cell therapy trials.

## Supplementary Materials

The following are available online at https://www.mdpi.com/article/10.3390/jcm10081792/s1, Figure S1: Neuronal cell-like properties of individual donor-derived TB-MSCs in comparison to BM-MSCs. TB-MSCs and BM-MSCs were compared for their response to growth factors and expression of receptors for NGF in three individual donors. (A) Response of TB and BM-MSCs to NGF. TB or BM-MSCs from three individual donors were stimulated by NGF (100 ng/ml) for 3 days, and their proliferative response in the presence or absence of NGF were quantified. Shown are the increments of cell numbers in the presence of NGF relative to the increase in absence of NGF in each individual donor (mean ± SEM, * *p* < 0.05, ** *p* < 0.01, *** *p* < 0.001, two expts, *n* = 3). (B) Response of TB and BM-MSCs to FGF. TB or BM-MSCs from three individual donors were stimulated by FGF (100 ng/ml) for 3 days. Shown are the increments of cell numbers in the presence of FGF relative to the increase in the absence of FGF in each individual donor (left) and mean of individual donors (B) (mean ± SEM, * *p* < 0.05, ** *p* < 0.01, *** *p* < 0.001, three expts, *n* = 3 for each experiment). Figure S2: Bioinformatics analysis of differential secretome of TB and BM-MSCs. (A) Upstream analysis transcriptional regulators that can explain the observed increase of the secreted proteins up-regulated in supernatants of TB-MSCs relative to that of BM-MSCs. The analysis was performed using IPA software (Z-score > 2.0). Shown are the predicted upstream signals and their target molecules identified in the secretome. (B) Analysis for disease or function prediction of secretome enriched in TB-MSCs in comparison to BM-MSCs. The analysis was performed by IPA software (Z-score > 1.5). Figure S3: Bioinformatic analysis of differences in the secretome of TB-MSCs and AD-MSCs. (A, B) Profiles of analysis for distinct secretome in up-stream regulators predictions (A) and biological function prediction (B). (C) Gene ontology of secreted proteins upregulated in TB-MSCs in comparison to AD-MSCs. Shown are the gene clusters of up-regulated proteins in supernatants of TB-MSCs compared to AD-MSCs along with counts of genes in each clusters (FDR < 0.02). (D) Upstream analysis transcriptional regulators that can explain the observed increase of the secreted proteins up-regulated in supernatants of TB-MSCs relative to that of AD-MSCs. The analysis was performed using IPA software (Z-score > 2.0). Figure S4: Effects of TB or BM-MSCs on metastasis of breast cancer cells. The mice with primary tumors were examined for metastasis in liver and lung by microscopy to count the number of metastatic foci and size (A, B): Representative micrographs of metastasis by H/E staining and measurement of their length in mice co-implanted with BM-MSCs (A) or TB-MSCs (B). Metastatic foci in low magnification images and their high magnification of the area indicated by box. Table S1: Primer sequences used for RT-QPCR analysis of transcripts in TB and BM-MSCs. Table S2: Gene ontology clusters for PCA analysis of TB-MSCs in comparison to BM or AD-MSCs.

## References

[1] Keating A. (2006). Mesenchymal stromal cells. Curr. Opin. Hematol..

[2] Pittenger M.F., Mackay A.M., Beck S.C., Jaiswal R.K., Douglas R., Mosca J.D., Moorman M.A., Simonetti D.W., Craig S., Marshak D.R. (1999). Multilineage potential of adult human mesenchymal stem cells. Science.

[3] Prockop D.J. (1997). Marrow stromal cells as stem cells for nonhematopoietic tissues. Science.

[4] Simões I.N., Boura J.S., dos Santos F., Andrade P.Z., Cardoso C.M., Gimble J.M., da Silva C.L., Cabral J.M. (2013). Human mesenchymal stem cells from the umbilical cord matrix: Successful isolation and ex vivo expansion using serum-/xeno-free culture media. Biotechnol. J..

[5] Giordano A., Galderisi U., Marino I.R. (2007). From the laboratory bench to the patient’s bedside: An update on clinical trials with mesenchymal stem cells. J. Cell. Physiol..

[6] Dezawa M., Hoshino M., Ide C. (2005). Treatment of neurodegenerative diseases using adult bone marrow stromal cell-derived neurons. Expert Opin. Biol. Ther..

[7] Gang E.J., Jeong J.A., Hong S.H., Hwang S.H., Kim S.W., Yang I.H., Ahn C., Han H., Kim H. (2004). Skeletal myogenic differentiation of mesenchymal stem cells isolated from human umbilical cord blood. Stem Cells.

[8] Horwitz E.M., Gordon P.L., Koo W.K., Marx J.C., Neel M.D., McNall R.Y., Muul L., Hofmann T. (2002). Isolated allogeneic bone marrow-derived mesenchymal cells engraft and stimulate growth in children with osteogenesis imperfecta: Implications for cell therapy of bone. Proc. Natl. Acad. Sci. USA.

[9] Jorgensen C., Gordeladze J., Noel D. (2004). Tissue engineering through autologous mesenchymal stem cells. Curr. Opin. Biotechnol..

[10] Magne D., Vinatier C., Julien M., Weiss P., Guicheux J. (2005). Mesenchymal stem cell therapy to rebuild cartilage. Trends Mol. Med..

[11] Krampera M., Glennie S., Dyson J., Scott D., Laylor R., Simpson E., Dazzi F. (2002). Bone marrow mesenchymal stem cells inhibit the response of naive and memory antigen-specific T cells to their cognate peptide. Blood.

[12] Le Blanc K., Tammik L., Sundberg B., Haynesworth S.E., Ringden O. (2003). Mesenchymal stem cells inhibit and stimulate mixed lymphocyte cultures and mitogenic responses independently of the major histocompatibility complex. Scand. J. Immunol..

[13] Kim D.W., Chung Y.J., Kim T.G., Kim Y.L., Oh I.H. (2004). Cotransplantation of third-party mesenchymal stromal cells can alleviate single-donor predominance and increase engraftment from double cord transplantation. Blood.

[14] Noort W.A., Kruisselbrink A.B., in’t Anker P.S., Kruger M., van Bezooijen R.L., de Paus R.A., Heemskerk M.H., Lowik C.W., Falkenburg J.H., Willemze R. (2002). Mesenchymal stem cells promote engraftment of human umbilical cord blood-derived CD34(+) cells in NOD/SCID mice. Exp. Hematol..

[15] Le Blanc K., Rasmusson I., Sundberg B., Gotherstrom C., Hassan M., Uzunel M., Ringden O. (2004). Treatment of severe acute graft-versus-host disease with third party haploidentical mesenchymal stem cells. Lancet.

[16] Ringden O., Uzunel M., Rasmusson I., Remberger M., Sundberg B., Lonnies H., Marschall H.U., Dlugosz A., Szakos A., Hassan Z. (2006). Mesenchymal stem cells for treatment of therapy-resistant graft-versus-host disease. Transplantation.

[17] Colter D.C., Sekiya I., Prockop D.J. (2001). Identification of a subpopulation of rapidly self-renewing and multipotential adult stem cells in colonies of human marrow stromal cells. Proc. Natl. Acad. Sci. USA.

[18] Kucia M., Halasa M., Wsoczynski M., Baskiewicz-Masiuk M., Zuba-Surma E., Machalinski B., Ratajczak M.Z. (2006). A novel population of Oct-4+ SSEA-1+ CXCR4+ CD34+ CD133+ Lin- CD45- very smal embryonic-like (VSEL) stem cells identified in human cord blood. Blood.

[19] Smith J.R., Pochampally R., Perry A., Hsu S.C., Prockop D.J. (2004). Isolation of a highly clonogenic and multipotential subfraction of adult stem cells from bone marrow stroma. Stem Cells.

[20] Lee G.Y., Jeong S.Y., Lee H.R., Oh I.H. (2019). Age-related differences in the bone marrow stem cell niche generate specialized microenvironments for the distinct regulation of normal hematopoietic and leukemia stem cells. Sci. Rep..

[21] Burja B., Barlič A., Erman A., Mrak-Poljšak K., Tomšič M., Sodin-Semrl S., Lakota K. (2020). Human mesenchymal stromal cells from different tissues exhibit unique responses to different inflammatory stimuli. Curr. Res. Transl. Med..

[22] Hass R., Kasper C., Böhm S., Jacobs R. (2011). Different populations and sources of human mesenchymal stem cells (MSC): A comparison of adult and neonatal tissue-derived MSC. Cell Commun. Signal..

[23] Kern S., Eichler H., Stoeve J., Klüter H., Bieback K. (2006). Comparative analysis of mesenchymal stem cells from bone marrow, umbilical cord blood, or adipose tissue. Stem Cells.

[24] Kim J.H., Lee H.S., Choi H.K., Kim J.A., Chu I.S., Leem S.H., Oh I.H. (2016). Heterogeneous Niche Activity of Ex-Vivo Expanded MSCs as Factor for Variable Outcomes in Hematopoietic Recovery. PLoS ONE.

[25] Hauser S., Widera D., Qunneis F., Müller J., Zander C., Greiner J., Strauss C., Lüningschrör P., Heimann P., Schwarze H. (2012). Isolation of novel multipotent neural crest-derived stem cells from adult human inferior turbinate. Stem Cells Dev..

[26] Tomé M., Lindsay S.L., Riddell J.S., Barnett S.C. (2009). Identification of nonepithelial multipotent cells in the embryonic olfactory mucosa. Stem Cells.

[27] Murrell W., Féron F., Wetzig A., Cameron N., Splatt K., Bellette B., Bianco J., Perry C., Lee G., Mackay-Sim A. (2005). Multipotent stem cells from adult olfactory mucosa. Dev. Dyn..

[28] Trentin A., Glavieux-Pardanaud C., Le Douarin N.M., Dupin E. (2004). Self-renewal capacity is a widespread property of various types of neural crest precursor cells. Proc. Natl. Acad. Sci. USA.

[29] Stemple D.L., Anderson D.J. (1992). Isolation of a stem cell for neurons and glia from the mammalian neural crest. Cell.

[30] Nagoshi N., Shibata S., Kubota Y., Nakamura M., Nagai Y., Satoh E., Morikawa S., Okada Y., Mabuchi Y., Katoh H. (2008). Ontogeny and multipotency of neural crest-derived stem cells in mouse bone marrow, dorsal root ganglia, and whisker pad. Cell Stem Cell.

[31] Jung J., Moon N., Ahn J.Y., Oh E.J., Kim M., Cho C.S., Shin J.C., Oh I.H. (2009). Mesenchymal stromal cells expanded in human allogenic cord blood serum display higher self-renewal and enhanced osteogenic potential. Stem Cells Dev..

[32] Wen H.C., Chuu C.P., Chen C.Y., Shiah S.G., Kung H.J., King K.L., Su L.C., Chang S.C., Chang C.H. (2015). Elevation of soluble guanylate cyclase suppresses proliferation and survival of human breast cancer cells. PLoS ONE.

[33] Shin S., Lee J., Kwon Y., Park K.S., Jeong J.H., Choi S.J., Bang S.I., Chang J.W., Lee C. (2021). Comparative Proteomic Analysis of the Mesenchymal Stem Cells Secretome from Adipose, Bone Marrow, Placenta and Wharton’s Jelly. Int. J. Mol. Sci..

[34] Shin J., Kwon Y., Lee S., Na S., Hong E.Y., Ju S., Jung H.G., Kaushal P., Shin S., Back J.H. (2019). Common Repository of FBS Proteins (cRFP) To Be Added to a Search Database for Mass Spectrometric Analysis of Cell Secretome. J. Proteome Res..

[35] Almagro Armenteros J.J., Tsirigos K.D., Sønderby C.K., Petersen T.N., Winther O., Brunak S., von Heijne G., Nielsen H. (2019). SignalP 5.0 improves signal peptide predictions using deep neural networks. Nat. Biotechnol..

[36] Bendtsen J.D., Jensen L.J., Blom N., Von Heijne G., Brunak S. (2004). Feature-based prediction of non-classical and leaderless protein secretion. Protein Eng. Des. Sel..

[37] Möller S., Croning M.D., Apweiler R. (2001). Evaluation of methods for the prediction of membrane spanning regions. Bioinformatics.

[38] da Silva Meirelles L., de Deus Wagatsuma V.M., Malta T.M., Bonini Palma P.V., Araújo A.G., Panepucci R.A., Silva W.A., Kashima S., Covas D.T. (2016). The gene expression profile of non-cultured, highly purified human adipose tissue pericytes: Transcriptomic evidence that pericytes are stem cells in human adipose tissue. Exp. Cell Res..

[39] Smyth L.C.D., Rustenhoven J., Scotter E.L., Schweder P., Faull R.L.M., Park T.I.H., Dragunow M. (2018). Markers for human brain pericytes and smooth muscle cells. J. Chem. Neuroanat..

[40] Boudjadi S., Chatterjee B., Sun W., Vemu P., Barr F.G. (2018). The expression and function of PAX3 in development and disease. Gene.

[41] Isern J., García-García A., Martín A.M., Arranz L., Martín-Pérez D., Torroja C., Sánchez-Cabo F., Méndez-Ferrer S. (2014). The neural crest is a source of mesenchymal stem cells with specialized hematopoietic stem cell niche function. eLife.

[42] Shafit-Zagardo B., Kalcheva N. (1998). Making sense of the multiple MAP-2 transcripts and their role in the neuron. Mol. Neurobiol..

[43] Gordon S.L., Leube R.E., Cousin M.A. (2011). Synaptophysin is required for synaptobrevin retrieval during synaptic vesicle endocytosis. J. Neurosci..

[44] Tarsa L., Goda Y. (2002). Synaptophysin regulates activity-dependent synapse formation in cultured hippocampal neurons. Proc. Natl. Acad. Sci. USA.

[45] Zucchi E., Bonetto V., Sorarù G., Martinelli I., Parchi P., Liguori R., Mandrioli J. (2020). Neurofilaments in motor neuron disorders: Towards promising diagnostic and prognostic biomarkers. Mol. Neurodegener..

[46] Mullen R.J., Buck C.R., Smith A.M. (1992). NeuN, a neuronal specific nuclear protein in vertebrates. Development.

[47] Murray L., Chen B., Galy A., Chen S., Tushinski R., Uchida N., Negrin R., Tricot G., Jagannath S., Vesole D. (1995). Enrichment of human hematopoietic stem cell activity in the CD34+Thy-1+Lin- subpopulation from mobilized peripheral blood. Blood.

[48] Péault B., Weissman I.L., Buckle A.M., Tsukamoto A., Baum C. (1993). Thy-1-expressing CD34+ human cells express multiple hematopoietic potentialities in vitro and in SCID-hu mice. Nouv. Rev. Fr. Hematol..

[49] Jeong S.Y., Kim J.A., Oh I.H. (2018). The Adaptive Remodeling of Stem Cell Niche in Stimulated Bone Marrow Counteracts the Leukemic Niche. Stem Cells.

[50] Kim J.A., Shim J.S., Lee G.Y., Yim H.W., Kim T.M., Kim M., Leem S.H., Lee J.W., Min C.K., Oh I.H. (2015). Microenvironmental remodeling as a parameter and prognostic factor of heterogeneous leukemogenesis in acute myelogenous leukemia. Cancer Res..

[51] Oh I.H., Kwon K.R. (2010). Concise review: Multiple niches for hematopoietic stem cell regulations. Stem Cells.

[52] Dittmer J., Rody A. (2013). Cancer stem cells in breast cancer. Histol. Histopathol..

[53] Velasco-Velázquez M.A., Popov V.M., Lisanti M.P., Pestell R.G. (2011). The role of breast cancer stem cells in metastasis and therapeutic implications. Am. J. Pathol..

[54] Czapla J., Matuszczak S., Kulik K., Wiśniewska E., Pilny E., Jarosz-Biej M., Smolarczyk R., Sirek T., Zembala M.O., Zembala M. (2019). The effect of culture media on large-scale expansion and characteristic of adipose tissue-derived mesenchymal stromal cells. Stem Cell Res. Ther..

[55] Sheng G. (2015). The developmental basis of mesenchymal stem/stromal cells (MSCs). BMC Dev. Biol..

[56] Kretlow J.D., Jin Y.Q., Liu W., Zhang W.J., Hong T.H., Zhou G., Baggett L.S., Mikos A.G., Cao Y. (2008). Donor age and cell passage affects differentiation potential of murine bone marrow-derived stem cells. BMC Cell Biol..

[57] Wagner W., Bork S., Horn P., Krunic D., Walenda T., Diehlmann A., Benes V., Blake J., Huber F.X., Eckstein V. (2009). Aging and replicative senescence have related effects on human stem and progenitor cells. PLoS ONE.

[58] Xin Y., Wang Y.M., Zhang H., Li J., Wang W., Wei Y.J., Hu S.S. (2010). Aging adversely impacts biological properties of human bone marrow-derived mesenchymal stem cells: Implications for tissue engineering heart valve construction. Artif. Organs.

[59] Viswanathan S., Keating A., Deans R., Hematti P., Prockop D., Stroncek D.F., Stacey G., Weiss D.J., Mason C., Rao M.S. (2014). Soliciting strategies for developing cell-based reference materials to advance mesenchymal stromal cell research and clinical translation. Stem Cells Dev..

[60] Squillaro T., Peluso G., Galderisi U. (2016). Clinical Trials With Mesenchymal Stem Cells: An Update. Cell Transplant..

[61] Zaim M., Karaman S., Cetin G., Isik S. (2012). Donor age and long-term culture affect differentiation and proliferation of human bone marrow mesenchymal stem cells. Ann. Hematol..

[62] Wagner W., Horn P., Castoldi M., Diehlmann A., Bork S., Saffrich R., Benes V., Blake J., Pfister S., Eckstein V. (2008). Replicative senescence of mesenchymal stem cells: A continuous and organized process. PLoS ONE.

[63] Anderson D.J. (1999). Lineages and transcription factors in the specification of vertebrate primary sensory neurons. Curr. Opin. Neurobiol..

[64] Korzh V., Strähle U. (2002). Proneural, prosensory, antiglial: The many faces of neurogenins. Trends Neurosci..

[65] Canel M., Serrels A., Frame M.C., Brunton V.G. (2013). E-cadherin-integrin crosstalk in cancer invasion and metastasis. J. Cell Sci..

[66] Lester B.R., McCarthy J.B. (1992). Tumor cell adhesion to the extracellular matrix and signal transduction mechanisms implicated in tumor cell motility, invasion and metastasis. Cancer Metastasis Rev..

[67] Paolillo M., Schinelli S. (2019). Extracellular Matrix Alterations in Metastatic Processes. Int. J. Mol. Sci..

[68] Stetler-Stevenson W.G., Aznavoorian S., Liotta L.A. (1993). Tumor cell interactions with the extracellular matrix during invasion and metastasis. Annu. Rev. Cell Biol..

