# Supplementary material for: Nasal Turbinate Mesenchymal Stromal Cells Preserve Characteristics of Their Neural Crest Origin and Exert Distinct Paracrine Activity
Source: J Clin Med. 2021 Apr 20;10(8):1792. doi: 10.3390/jcm10081792 (PMC8074274; doi:10.3390/jcm10081792)
Supplement: Supplementary file 1 [file jcm-10-01792-s001.zip › jcm-1176224-supplementary.pdf]

Journal of
Clinical Medicine

MDPI

Article

Supplementary Material: Nasal Turbinate Mesenchymal Stromal Cells Preserve Characteristics of Their Neural Crest Origin and Exert Distinct Paracrine Activity

Hyun-Jee Kim, Sungho Shin, Seon-Yeong Jeong, Sun-Ung Lim, Dae-Won Lee, Yunhee-Kim Kwon, Jiyeon Kang, Sung-Won Kim, Chan-Kwon Jung, Cheolju Lee and Il-Hoan Oh

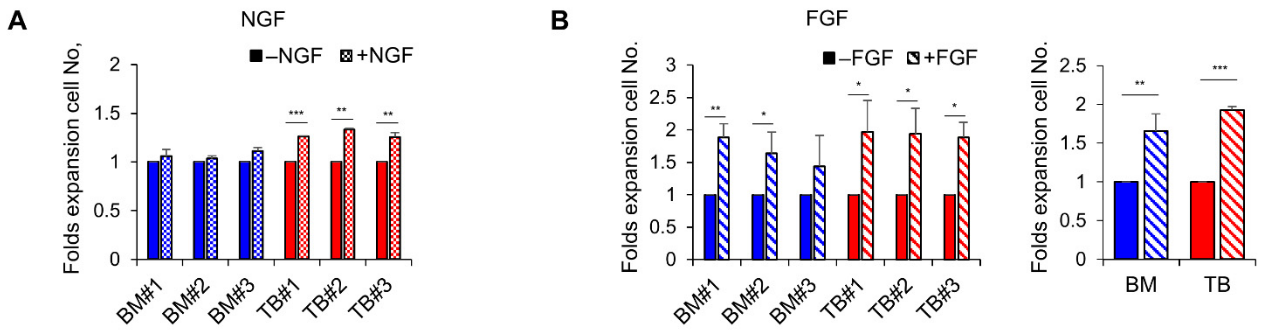

Figure S1. Neuronal cell-like properties of individual donor-derived TB-MSCs in comparison to BM-MSCs. TB-MSCs and BM-MSCs were compared for their response to growth factors and expression of receptors for NGF in three individual donors. (**A**) Response of TB and BM-MSCs to NGF. TB or BM-MSCs from three individual donors were stimulated by NGF (100 ng/ml) for 3 days, and their proliferative response in the presence or absence of NGF were quantified. Shown are the increments of cell numbers in the presence of NGF relative to the increase in absence of NGF in each individual donor (mean ± SEM, $*p < 0.05$, $**p < 0.01$, $***p < 0.001$, two expts, $n = 3$). (**B**) Response of TB and BM-MSCs to FGF. TB or BM-MSCs from three individual donors were stimulated by FGF (100 ng/ml) for 3 days. Shown are the increments of cell numbers in the presence of FGF relative to the increase in the absence of FGF in each individual donor (left) and mean of individual donors (B) (mean ± SEM, $*p < 0.05$, $**p < 0.01$, $***p < 0.001$, three expts, $n = 3$ for each experiment).

A

TB-BM upstream analysis			
Upstream Regulator	Activation Z-score	Molecule Type	Target Molecules in Dataset
NEUROG1	3.00	transcription regulator	C3,CXCL1,IL6,INHBA,MFAP4,P4HA2,PAPPA,PPIC,S100A4
HMOX1	2.19	enzyme	CXCL1,CXCL3,CXCL5,IL6,VEGFA
CBX5	2.00	transcription regulator	CPA4,CXCL5,QPCT,TGFBI
HIC1	2.00	transcription regulator	FHL2,INHBA,LIF,SRGN
WT1	2.00	transcription regulator	APLP2,CMPK1,CSF1,HSP90B1,VEGFA
MYC	2.00	transcription regulator	ALCAM,AXL,CD44,COL1A1,SERPINE2,TMSB10/TMSB4X,VEGFA

B

TB-BM disease and function predict			
Categories	Diseases or Functions Annotation	Activation Z-score	*p*-Value
Cellular Development, Cellular Growth and Proliferation	Proliferation of prostate cancer cell lines	2.172	0.00001
Cellular Movement, Hematological System Development and Function, Immune Cell Trafficking, Inflammatory Response	Transmigration of phagocytes	1.951	0.00021
Cellular Movement, Hematological System Development and Function, Immune Cell Trafficking, Inflammatory Response	Migration of phagocytes	1.856	0.00000
Cellular Movement, Hematological System Development and Function, Immune Cell Trafficking, Inflammatory Response	Migration of neutrophils	1.715	0.00015
Cell-To-Cell Signaling and Interaction	Binding of leukemia cell lines	1.689	0.00022
Cell Death and Survival	Cell death of neuroglia	1.686	0.00002
Cell-To-Cell Signaling and Interaction, Cellular Assembly and Organization	Cell-cell contact	1.675	0.00002

Figure S2. Bioinformatics analysis of differential secretome of TB and BM-MSCs. (**A**) Upstream analysis transcriptional regulators that can explain the observed increase of the secreted proteins up-regulated in supernatants of TB-MSCs relative to that of BM-MSCs. The analysis was performed using IPA software (Z-score > 2.0). Shown are the predicted upstream signals and their target molecules identified in the secretome. (**B**) Analysis for disease or function prediction of secretome enriched in TB-MSCs in comparison to BM-MSCs. The analysis was performed by IPA software (Z-score > 1.5).

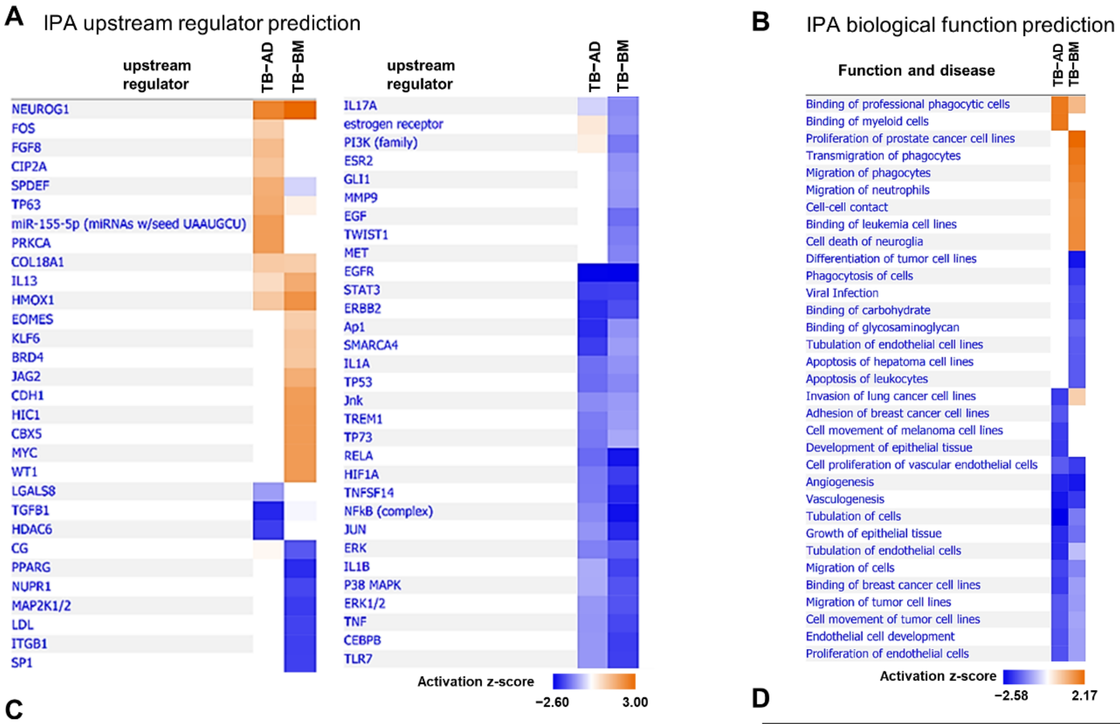

C

TB up cluster		
Gene ontology biological process	Count	FDR
cell adhesion	8	0.00522
regulation of cell growth	4	0.06845

D

TB-AD_upstream analysis	
Upstream Regulator	Activation z-score
NEUROG1	2.45
Ap1	- 2.17
EGFR	- 2.59
TGFB1	- 2.22
ERBB2	- 2.16

Figure S3. Bioinformatic analysis of differences in the secretome of TB-MSCs and AD-MSCs. (**A**,**B**) Profiles of analysis Figure 0. (**C**,**D**) Upstream analysis transcriptional regulators that can explain the observed increase of the secreted proteins up-regulated in supernatants of TB-MSCs relative to that of AD-MSCs. The analysis was performed using IPA software (Z-score > 2.0).

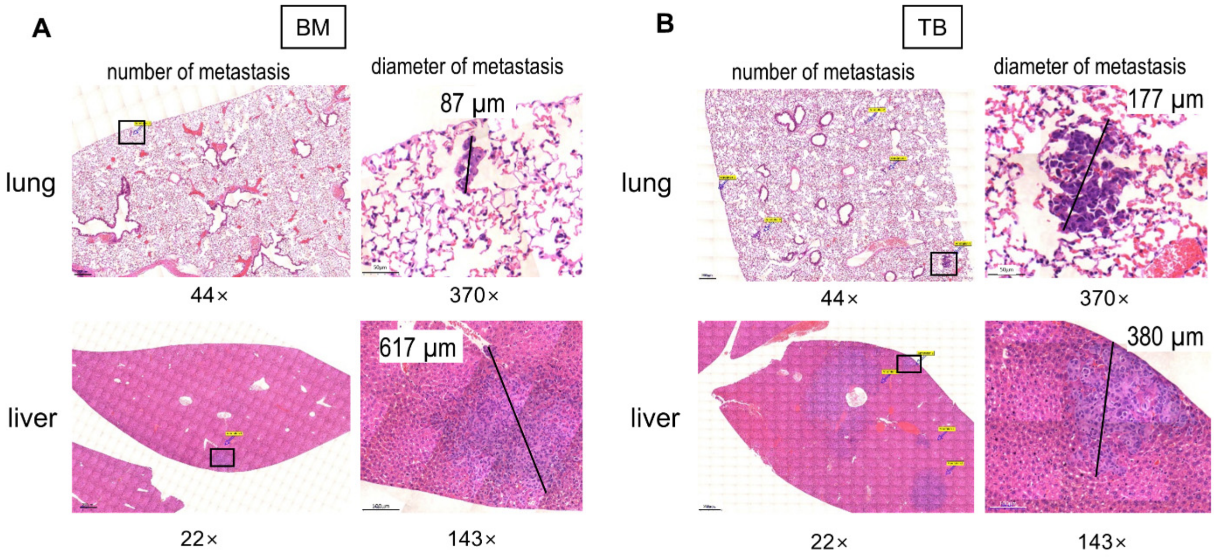

Figure S4. Effects of TB or BM-MSCs on metastasis of breast cancer cells. The mice with primary tumors were examined for metastasis in liver and lung by microscopy to count the number of metastatic foci and size (**A**,**B**): Representative micrographs

of metastasis by H/E staining and measurement of their length in mice co-implanted with BM-MSCs (**A**) or TB-MSCs (**B**). Metastatic foci in low magnification images and their high magnification of the area indicated by box.

Table S1. Primer sequences used for RT-QPCR analysis of transcripts in TB and BM-MSCs.

**Gene Name**		**5'–3'**
NESTIN	Forward	CAG CGT TGG AAC AGA GGT TGG
	Reverse	TGG CAC AGG TGT CTC AAG GGT AG
NG2	Forward	GCT TTG ACC CTG ACT ATG TTG
	Reverse	TCC AGA GTA GAG CTG CAG CA
CD146	Forward	AAG GCA ACC TCA GCC ATG TCG
	Reverse	CTC GAC TCC ACA GTC TGG GAC
PDGFRβ	Forward	CAG TAA GGA GGA CTT CCT GGA G
	Reverse	CCT GAG AGA TCT GTG GTT CCA G
PAX3	Forward	GCA CTG TAC ACC AAA GCA CG
	Reverse	TAG GTG GGT GGA CAG TAG GA
CD13	Forward	GGC CTT CAT TGT CAG TGA GT
	Reverse	TCT GAT TTT GGG AGT GGG TA
SOX2	Forward	AGC TAC AGC ATG ATG CAG GA
	Reverse	GGT CAT GGA GTT GTA CTG CA
OCT4	Forward	CGA GCA ATT TGC CAA GCT CCT GAA
	Reverse	TTC GGG CAC TGC AGG AAC AAA TTC
NANOG	Forward	CAA AGG CAA ACA ACC CAC TT
	Reverse	TCT GCT GGA GGC TGA GGT AT
MAP2	Forward	CCA AGC GGC TAC ACG TCT
	Reverse	GCT CGG TCA GCA TCT GAG
SYP	Forward	TGC CAA CAA GAC CGA G
	Reverse	TGC CGA TGA GCT AAC T
PAX3	Forward	GCA CTG TAC ACC AAA GCA CG
	Reverse	TAG GTG GGT GGA CAG TAG GA

NG2, Neuron-glial antigen 2; *PDGFRβ*, Platelet Derived Growth Factor Receptor Beta; *PAX3*, paired box gene 3; *SOX2*, SRY-Box Transcription Factor 2; *OCT4*, also known as POU5F1 (POU domain class 5 transcription factor 1); *MAP2*, Microtubule Associated Protein 2; *SYP*, synaptophysin.

Table S2. Gene ontology clusters for PCA analysis of TB-MSCs in comparison to BM or AD-MSCs.

PC1 positive 25%			PC2 positive 25%		
**Gene Ontology Biological Process**	**Count**	**FDR**	**Gene Ontology Biological Process**	**Count**	**FDR**
extracellular matrix organization	16	0.00000	cell-cell adhesion	9	0.01020
cell adhesion	13	0.00015	gluconeogenesis	5	0.01030
platelet degranulation	7	0.00116	canonical glycolysis	4	0.03480
collagen fibril organization	5	0.00405			
collagen catabolic process	5	0.02320			
glycosaminoglycan metabolic process	4	0.03020			
skeletal system development	6	0.03020			
**PC1 Negative 25%**			**PC2 Negative 25%**		
**Gene Ontology Biological Process**	**Count**	**FDR**	**Gene Ontology Biological Process**	**Count**	**FDR**
extracellular matrix organization	14	0.00000	extracellular matrix organization	26	0.00000
cell adhesion	14	0.00002	cell adhesion	22	0.00000
platelet degranulation	7	0.00113	collagen fibril organization	10	0.00000
negative regulation of endopeptidase activity	7	0.00214	skeletal system development	10	0.00000
collagen fibril organization	5	0.00315	collagen catabolic process	8	0.00000
cellular protein metabolic process	6	0.01700	platelet degranulation	7	0.00046
endodermal cell differentiation	4	0.02080	extracellular matrix disassembly	6	0.00144
angiogenesis	7	0.02950	protein heterotrimerization	4	0.00193
wound healing	5	0.02950	osteoblast differentiation	6	0.00511
response to estradiol	5	0.03920	cellular protein metabolic process	6	0.00836
negative regulation of apoptotic process	9	0.03920	endodermal cell differentiation	4	0.01090
aging	6	0.03920	collagen biosynthetic process	3	0.01340
platelet aggregation	4	0.03920	angiogenesis	7	0.01610
cell migration	6	0.04140	blood vessel development	4	0.02380
actin crosslink formation	3	0.04450	cellular response to amino acid stimulus	4	0.04160
cell-cell adhesion	7	0.04450			
